# Prospective, Randomized, Double-Blind Parallel Group Nutritional Study to Evaluate the Effects of Routine Intake of Fresh vs. Pasteurized Yogurt on the Immune System in Healthy Adults

**DOI:** 10.3390/nu16121969

**Published:** 2024-06-20

**Authors:** Fernando Rivero-Pino, Mar Casquete, Maria José Castro, Paz Redondo del Rio, Eloina Gutierrez, Agustín Mayo-Iscar, Mercedes Nocito, Alfredo Corell

**Affiliations:** 1 Department of Medical Biochemistry, Molecular Biology, and Immunology, School of Medicine, University of Seville, 41009 Seville, Spain; 2 Instituto de Biomedicina de Sevilla, IBiS, Hospital Universitario Virgen del Rocío, CSIC, University of Seville, 41013 Seville, Spain; 3 Departamento de Pediatría, Inmunología, Obstetricia-Ginecología, Nutrición-Bromatología, Universidad de Valladolid, 47005 Valladolid, Spain; 4 Departamento de Enfermería, Universidad de Valladolid, 47003 Valladolid, Spain; 5 Departamento de Estadística e Investigación Operativa & IMUVA, Universidad de Valladolid, 47011 Valladolid, Spain; 6 Inmunología, Hospital Clínico de Zaragoza, 50009 Zaragoza, Spain

**Keywords:** *Lactobacillus delbrueckii* subsp. *bulgaricus*, gut microbiota, probiotics, *Streptococcus thermophilus*, inactivated bacteria, alive bacteria

## Abstract

The immune system is affected by the dietary products humans intake. Immune system regulation by nutrition has uses in the clinical context, but it can also benefit healthy populations by delaying or preventing the emergence of immune-mediated chronic illnesses. In this study, the purpose was to describe and compare the modulator effects on the immune system of the routine ingestion of fresh vs. pasteurized yogurt. A unicentral, prospective, randomized, double-blind, parallel group 8-week nutritional study was carried out comparing the ingestion of 125 g of the products in healthy adults three times a day. A complete battery of in vitro tests on the activity of the immune system, processes and phenomena was performed. Exclusive immune-modulatory effects of fresh yogurt with respect to base line were found in terms of increased systemic IgM (primary immune responses), increased synthesis of IFN-gamma upon stimulation (Th1) and increased peripheral T cells (mainly “naive” CD4s). In the three interventions, we observed an increased phagocytic activity and burst test in granulocytes, together with increased secretion of IL-6, IL-1 β and IL-8 (pro-inflammatory) and increased CD16 expression (FcR favoring phagocytosis) in granulocytes. Overall, it is concluded that regardless of bacteria being alive or thermally inactivated, yogurt has common effects on the innate system, but the presence of live bacteria is necessary to achieve a potentiating effect on the specific immune response.

## 1. Introduction

Our immune system needs to be able to offer robust and sufficient protection against invading pathogens while tolerating dietary proteins and commensal bacteria since eating exposes us to nearly continuous and significant antigenic stimulation [1]. A well-balanced diet should provide sufficient nutrients to adequately meet metabolic demands and promote a sense of wellness. However, emerging evidence supports the hypothesis that diet modulates specific biological functions within the body, conferring additional physiological and psychological benefits beyond the well-documented nutritional effects. Therefore, in addition to supporting growth and maintaining overall health, nutrition may play a crucial role in mitigating the risk of various diseases [2].

The human gastrointestinal tract hosts approximately 400 species of bacteria that maintain a symbiotic relationship with the individual [3]. Bacterial colonization of the intestine starts immediately after birth and persists throughout the entire life, showing relevant age-dependent changes. Under normal conditions, bacteria living within the intestine do not cause acute harmful effects, and some of them have been shown to be relevant for an adequate host’s homeostasis [4]. This microbiota maintains an intimate relationship with the host’s immune system, acting towards pathogen microorganisms, actively producing antimicrobials, and entailing the host’s adaptations to adequately react against external pathogens and external injuries by both the improvement of defensive mechanisms in the gastrointestinal tract and the modulation of the immune system to expand its response to several stimuli [5]. An appropriate intervention over intestinal microbiota could, therefore, translate into health benefits. In this way, its deliberate modification by the ingestion of probiotics is gaining traction as the amount of scientific evidence of the associated potential health benefits increases [3].

The basic and clinical studies that have been performed with lactic acid bacteria used in the production of fermented dairy products represent one of these sources of evidence. The ingestion of fermented milk has been shown to transitorily modify the intestinal microbiota because of the survival of external species. Thus, yogurt ingestion may increase the amount of lactic acid bacteria at different levels of the human intestine [6,7] and modify the functions of the intestinal microbiota. The presence of lactobacilli in the diet was associated with a reduction in human fecal enzymatic activity of beta-glucuronidase, nitroreductase, and glycocholic acid reductase and the urinary excretion of p-cresol, among other changes in bacterial enzymes. Some studies have revealed that the benefits of ingesting fermented dairy products (e.g., yogurt instead of milk) was more beneficial in children with diarrhea, decreasing the proportion of clinical failures [8,9,10].

The immunostimulant effects of lactic acid bacteria and yogurt are attributed to the intestinal microecology, and are dependent on contact with the intestine-associated lymphoid tissue during bacterial colonization of the mucosa [11]. Pasteurized yogurts are available in the market, and it seems reasonable to expect that the benefits associated to the presence of viable bacteria in yogurt would not be present if the product was pasteurized, as it has been reported in some clinical trials [8,9,12]. According to the published literature, with the exception of some small studies [6,13], the studies that have compared the benefits associated with the routine intake of fresh versus pasteurized yogurt have been limited to the assimilation of nutrients, oral cecal transit time, survival of probiotics or changes in fecal microbiota. There is preliminary evidence that immunostimulation by fresh yogurt is associated with a decreased incidence of certain tumors, gastrointestinal disorders, and allergic conditions [11]. In different in vitro studies, different Lactobacillus species have demonstrated that when alive, they could promote the secretion of TNF-α, IL-1β, IL-6 and IFN-γ, unlike in the case of heat-killed Lactobacillus, which did not show the capacity to promote the secretion of IL-1β and TNF-α [14].

To evaluate the biological basis of these clinical observations, the present study aimed to perform a comparison in terms of short-term immune system modulation. The objective of this study was to describe the modulator effects on the immune system of the bacteria contained in yogurt, either alive or thermally deactivated, to elucidate how these bacteria might impact different levels of the immune system and confer the adults’ specific immune responses.

## 2. Materials and Methods

### 2.1. Diagnosis and Main Selection Criteria

The criteria for the screening visit included the following requirements: Healthy subjects (absence of any chronic pharmacotherapy) aged between 20 and 70 years old and with a body mass index (BMI) between 18.5 and 29.9 kg/m^2^, who were following a balanced diet without engagement in any therapeutic lifestyle changes involving stringent dietary interventions such as weight-reducing diets. A minimum requirement of 42 evaluable subjects in each of the interventional groups (fresh and pasteurized yogurt) was established to detect differences of at least 60%, the common standard deviation of any parameter accepting parametric description with a statistical power of 80% at a bilateral 5% significance level. Clinical biochemistry and hematology parameters were measured as part of the eligibility criteria. In order to understand changes according to age, stratification was carried out in the ranges of 30–45–60 and older, and also according to gender. The study was conducted according to the Good Practice Guidelines and in line with the principles outlined in the Declaration of Helsinki of the World Medical Association. This study was approved by the Clinical Research Ethics Committee of the University of Valladolid (code YASI-03), on 15 April 2016, and written informed consent was obtained from all participants before entering the study. The information in relation to the stratification is not meant to be published but it may be available upon request.

### 2.2. Study Products

The study products (in 125 g pots) employed in this nutritional intervention were:

1. Product A: pasteurized yogurt. Pasteurized (heat-treated) natural yogurt containing < 15 CFU/g of *Lactobacillus delbrueckii* subsp. *bulgaricus* and <15 CFU/g of *Streptococcus thermophilus*. The product did not contain any other viable bacteria.

2. Product B: test product. Fresh natural yogurt containing ≥ 10^8^ CFU/g of *Lactobacillus delbrueckii* subsp. *bulgaricus* and ≥10^8^ CFU/g of *Streptococcus thermophilus*. The product did not contain any other viable bacteria.

3. Product C: sterilized yogurt. Sterilized (heat-treated) natural yogurt not containing any viable bacteria.

### 2.3. Nutritional Intervention

**Figure 1:**
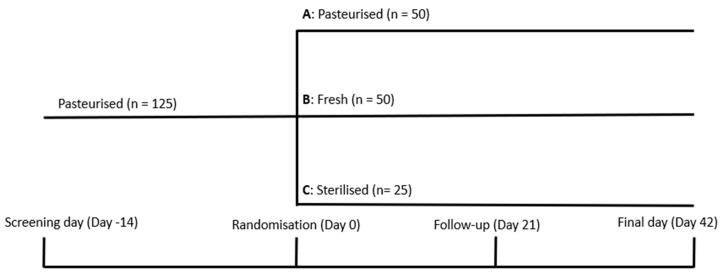
Design and periods of the study.

Subjects meeting the selection criteria entered a 2-week run-in period in which all patients received 125 g three times a day (tid) of pasteurized yogurt. At the end of this period, subjects meeting the relevant criteria were randomized to either switch to fresh yogurt (125 g tid), continue with pasteurized yogurt (125 g tid), or switch to sterilized yogurt (125 g) in an asymmetrical allocation of 2:2:1 (Figure 1). Additionally, a blood sample was taken to perform the panel of baseline immune tests (Section 2.4). Study products were taken for the subsequent 6 weeks. A follow-up visit was performed 3 weeks after the randomization and the final visit at the end of the 6-week supplementation period. Blood samples were also taken at the follow-up and final visits to perform the on-treatment panels of immune tests. The intake of any fermented dairy food other than study products was not allowed throughout the entire study period. In Figure 1, the schema of the intervention is shown. 

### 2.4. Measurement of Immune System Function Markers

#### 2.4.1. Leukocyte Subpopulations

A complete panel of leukocyte subpopulations was described over whole blood treated with EDTA using a wide range of mAbs combined with different fluorescent dyes for the flow cytometer. Tests included the measures of lymphocytes T total, T helper, T cytotoxic, with differentiation of naïve and memory cells; lymphocytes B and lymphocytes NK; lymphocytes, monocytes, and granulocytes CD11b+, HLA-DR+, and both. Some derived quotients were also calculated and included in the results. All results were expressed as cell number per microliter of peripheral blood and a percentage of total leukocytes [15].

Immunophenotype of lymphocytes was analyzed by flow cytometry. EDTA whole blood was tested in five separate labelling tubes. The flow cytometry analysis was performed with a Cytomics FC 500 Cytometer (Beckman-Coulter, Fullerton, CA, USA) using 488 nm excitation with an argon-ion laser for fluorescein isothiocyanate (FITC), phycoerythrin (PE), phycoerythrin-Texas Red-X (ECD), propidium iodide, phycoerythrin-cyanine 5 (PC5), and phycoerythrin-cyanine 7 (PC7). The data collected were analyzed using the Cytomics RXP software program (Beckman-Coulter). Controls included cross reactivity of the fluorescence signals of each channel, as well as isotype-matched unspecific monoclonal antibodies used as negative controls. Flow cytometry gates were applied as appropriate to analyze the cell phenotype.

The phenotype of peripheral leukocytes was determined by staining with five panels of fluorochrome-conjugated mouse anti-human monoclonal antibodies: tube 1 consisted of the mixture of anti-CD3−PC5/CD4−PE/CD8−ECD/CD 45FITC; tube 2: anti-CD3−PC5/CD56−PE+CD16−PE/CD19−ECD/CD45FITC; tube 3: mixed anti-CD45RA−PE/CD45RO−FITC/CD3−PC5/CD4−ECD; tube 4: mixed anti-CD45−PC7/CD11−PE/HLA−DR−FITC; and finally, tube 5: mixed anti-CD45−PC7/CD86−PE/CD14−FITC. All antibodies used in this panel were obtained from Beckman-Coulter. 

Cell suspensions were incubated in the dark with these antibodies at room temperature for 15 min. They were then incubated in the dark with 0.5 mL of FACS Lysing solution (BD Biosciences) at room temperature for 15 min to fix the cells. Afterwards, the cells were gently agitated, and flow cytometry analysis was performed.

#### 2.4.2. Immunochemistry

Serum immunoglobulins G, A, M, and E, the complement C3 factor, and C-reactive protein were also determined using Kinetic nephelometry (Dade Behring BNII) [16].

#### 2.4.3. Phagocytosis and Burst

The Phagotest and Bursttest kits from Orpegen Pharma were used to measure the ingestion of bacteria and oxidative burst, respectively, by whole blood using the manufacturer’s protocols [17]. The former uses fluorescein-labeled opsonized *Escherichia coli* that is measured after removing bacteria connected to the leukocyte surface and subsequent fixing of leukocytes. The latter uses unlabeled opsonized *E. coli* and oxidative burst is measured by the amount of non-fluorescent dihydrorhodamine transformed into fluorescent rhodamine. In both cases, fluorescence is measured in a Cytomics FC 500 Cytometer (Beckman-Coulter, Fullerton, CA, USA). Both procedures include a negative control. Surrogate indices of effect were calculated as the quotients between the results of test cultures and negative controls. 

#### 2.4.4. NK Cytotoxic Activity

The standard NK cell cytotoxic activity on peripheral blood mononuclear cells was determined using a LIVE/DEAD Viability/Cytotoxicity Assay Kit with two-color fluorescence based on the simultaneous determination of live and dead cells by using Calcein AM and ethidium propide. The staining of cells was performed using the manufacturer’s protocols (Molecular Probes, Eugene, OR, USA). Two effect-to-target ratios of 5:1 and 10:1 were considered to express the in vitro raw activity. The results were analyzed in a Cytomics FC 500 Cytometer (Beckman-Coulter, Fullerton, CA, USA) as previously described [18].

#### 2.4.5. IFN-γ Inducible Gene Expression

The expression of interferon-sensitive genes (2′,5′-oligoadenylate synthetase and toll-like receptors 7 to 9) was evaluated in whole blood by counting the copies of mRNA by quantitative real-time PCR of cDNA obtained through the reverse-transcription of mRNA [19]. Total cellular RNA was extracted using the RNeasy mini kit (Qiagen, Venlo, The Netherlands), and used as a template in reverse transcription reactions (RT) carried out using MMLV reverse transcriptase (Promega, Madison, WI, USA) and oligo dT as described by the manufacturer’s protocol. Quantitative PCRs were performed in a Roche Lightcycler 2.0 (Hoffmann-La Roche, Basel, Switzerland). The results are expressed as the number of transcripts per volume of cDNA dilute and normalized by the number of transcripts per copy of the β-actin gene. Information about the primers employed can be found in the literature [20,21].

#### 2.4.6. Intracellular Cytokine Profile

These tests were performed with unstimulated and stimulated peripheral whole blood with phytohemagglutinin (PHA) or anti-CD3+ anti-CD28 mAbs. Functional status (with the addition of Brefeldin A solution) and activation was determined with the FastImmune IFN-g/CD69/CD4/CD3 cytokine system reagent (BD, New Jersey, NJ, USA), following the manufacturer’s protocols. Counts were performed with a Cytomics FC 500 Cytometer (Beckman-Coulter, Fullerton, CA, USA), after treating the samples with FastImmune Lysing solution. Surrogate indices of effect were calculated as the quotients between the results of the stimulated and unstimulated samples.

#### 2.4.7. Cytokine Production

These tests were performed with and without the stimulation of peripheral blood cells with PHA or PHA or anti-CD3+ anti-CD28 mAbs. The FlowCytomix multiplex fluorescent bead immunoassay system was used to stain the analytes (TH1 and TH2 cytokines), following the manufacturer’s protocol (eBioscience, San Diego, CA, USA), and the concentrations were measured in a Cytomics FC 500 Cytometer (Beckman-Coulter, Fullerton, CA, USA) [22]. Surrogate indices of effect were calculated as the quotients between the results of the stimulated and unstimulated samples. 

### 2.5. Statistical Analysis

All variables were described by means of moment- and range-based measures (all variables were quantitative, with the exception of gender). Analyses were performed primarily on the per-protocol population, which comprised all randomized patients, because no major protocol violations occurred. 

Derived endpoints (i.e., the surrogate indices of activity) were calculated from the original values recorded in the database. Null activity values in control samples were imputed with the 10^−n^ value (where n is the number of decimals of the minimum value plus one) to avoid divisions by zero in the calculation of these indices. 

The data were analyzed using the Shapiro–Wilk test to determine the normal distribution. Absolute and relative changes from baseline were calculated for all variables and were compared among supplementation groups by means of analysis of variance of Kruskall–Wallis tests if the data did not admit parametric description. In cases where the parametric description was accepted, an additional analysis of covariance was adjusted to account for baseline differences among groups, and post hoc Tukey tests were run to test the pairwise differences between groups. 

## 3. Results and Discussion

### 3.1. Baseline

There were not any significant differences among groups in the distribution of the parameters shown in the Supplementary Materials (Tables S1 and S2), including routine biochemistry and hematology. In general, all of the aforementioned parameters were within normal ranges in all subjects, except for blood pressure and serum lipids, because some individuals were in the range of mild hypertension and/or showed mild to moderate dyslipidemia, that were considered irrelevant for the purposes of the study. There was one subject with eosinophilia, but this was not considered to significantly affect the outcomes. Seven subjects did not finish the intervention for not following the instructions.

### 3.2. Leukocyte and Lymphocyte Subpopulations

As it has been previously described, changes in the gut microbiota may influence the activity and composition of immune cells, including lymphocytes and leukocytes. In the case of yogurt, the live beneficial bacteria of the products containing it could have a different effect than the pasteurized product. However, these changes are likely to occur gradually and may depend on various factors, such as the specific strains of probiotics in the yogurt, the individual’s baseline health, and the overall diet [23]. To the authors’ knowledge, there is not a wealth of research specifically examining the direct and immediate effects of yogurt consumption on lymphocyte and leukocyte subpopulations.

As can be observed in Table 1, in relation to total T lymphocytes (CD3), individuals with C supplementation showed a slight decrease in circulating T lymphocytes, which reached sufficient significance at the end of the study. The stratification showed that this affects men of all ages and women under 45 years of age (data available upon request). In the case of Helper T lymphocytes (CD3 and CD4), when the change seen in total lymphocytes is studied in this majority subpopulation, the same trend is seen. Supplementation with products A and B induces an increase in T helper cells while supplementation with C induces a reduction. Both variations reach statistical significance. CD3 counts are indicative of the total T cell population, including both helper (CD4+) and cytotoxic (CD8+) T cells. A decrease in CD3 counts could be associated with various conditions affecting T cell function [24]. Nonetheless, cytotoxic T lymphocytes (CD3 CD8) show the opposite trend to the above. Individuals supplemented with product B showed a reduced percentage of cytotoxic T lymphocytes, which reached significance at the end of the study. On the other hand, the CD4/CD8 proportion is considered one of the best immune biomarkers of nutritional status, as it is modulated in situations of malnutrition. As a result of the alterations observed in both subpopulations, there is a significant increase in said ratio in the individuals who consumed product B. In the stratification, it was observed that the increase occurred mainly in women over 30 years of age (data available upon request). In a healthy immune system, the normal CD4/CD8 ratio is typically greater than 1, generally from 1.5 to 2.5 [25], which is in the range of the values obtained in this study.

In relation to the parameters evaluated, many of them did not show any statistically significant differences, including minority populations of T cells: CD4, CD8 double-negative or double-positive, as well as CD3+ CD56+ cells. The three supplementations induced a reduction in CD3-negative CD8-positive cells (subpopulation of NK lymphocytes) in a similar way without differences between yogurts that have been consumed. However, it must be noted that a significant increase was reported in B lymphocytes in the individuals who consumed product C. The CD3/CD19 ratio is another indicator of nutritional status. A clear tendency to decrease is seen in individuals who consumed product C, which is logical since it is parallel to the decrease in T lymphocytes and the increase in B lymphocytes. However, this trend does not reach sufficient statistical significance. The CD3/CD19 ratio reflects the balance between T cells and B cells, two major components of the adaptive immune system. Maintaining a proper balance between these cell types is crucial for overall immune function [26].

Regarding NK lymphocytes (CD3-negative CD16 and/or CD56-positive), individuals who consumed products A and B suffered a significant decrease in their circulating NK cells, while those who consumed product C also suffered a significant increase. In the stratification, it was observed that this increase occurred in the age group between 20 and 45 years, affecting both genders equally (data available upon request). In the case of naïve T lymphocytes (CD3+ CD45RA+ R0−), there is a clear trend toward an increase in this subpopulation in the individuals who consumed product B and a decrease in the individuals who consumed product C but without statistical significance. On the contrary, memory T lymphocytes can be seen to act the opposite compared to the previous subpopulation, with a clear increase in memory T lymphocytes in the individuals who consumed product C but without statistical significance. Regarding the CD45RA/R0 CD3 ratio, the index, in this case, accentuates the differences in both parameters with a decrease in the individuals who consumed product C but without reaching significance. No significant differences are seen in the expression density of these molecules in T cells or in the double-negative or double-positive subpopulations (for CD45RA and CD45R0). For naïve T helper lymphocytes, the trend is identical to that observed in total T lymphocytes but without statistical significance.

No significant differences are seen regarding the memory helper T cells, nor in the expression density of these molecules, in the double-positive or double-negative populations, or in the CD45RA/RO ratio within the T helper lymphocytes. For naïve cytotoxic T lymphocytes, a slight reduction was reported in group C, but it was not significant. For the cytotoxic memory T lymphocytes: individuals who consumed product B saw them decrease, while those who consumed product C saw them increase, as was the case in total T-, but again, these differences were not statistically significant. No significant differences are seen in the expression density of these molecules, in the double-positive or double-negative populations, nor in the CD45RA/R0 ratio within the cytotoxic T lymphocytes.

Regarding the CD16 expression in granulocytes, for the three supplementation groups, an increase in the number of cells expressing (achieving 99% in all cases) CD16 and a slight decrease in the density of the molecules per cell is observed (from 307.41 ± 97.35 to 282.08 ± 86.06, 293.52 ± 79.16 and 278.18 ± 92.65 for A, B, and C, respectively, measured as mean intensity of fluorescence, MFI). Both changes are significant. CD16 expression on immune cells, such as natural killer cells and macrophages, is crucial for mediating antibody-dependent cellular cytotoxicity, enhancing the immune system’s ability to eliminate target cells, and contributing to defense against infections and tumor cells [27].

Individuals who consumed product C showed a non-significant increase in HLA-DR expression on lymphocytes and a reduction in the density of DR per lymphocyte, which did reach significance with respect to the baseline. On the other hand, in monocytes and granulocytes, there are no significant differences or clear trends in the DR expression profile. The relevance of HLA-DR expression in the context of nutrition is primarily associated with its role in immune function and the modulation of adaptative immune responses because it is the main antigen-presenting protein to T helper cells. Nutrition plays a crucial role in supporting overall immune health, and certain nutrients can influence the expression and function of immune-related molecules, including HLA-DR [28]. In the same line as this study, Meng et al. [29] demonstrated the benefits of *Bifidobacterium animalis* subsp. lactis BB-12 at a dose of log10 ± 0.5 CFUs/day on immune responses in a randomized, partially blinded, four-period crossover, free-living study. These authors reported an increase in the percentage of CD14+HLA-DR+ cells in peripheral blood as well as a significantly lower expression of TLR-2 on CD14+HLA-DR+ cells and a reduction in TNF-α secretion from BB-12- or LPS-stimulated PBMCs compared to baseline.

Considering the CD11b expression involved in the recognition and binding of cells to sites of inflammation, facilitating the clearance of pathogens and cellular debris, which is relevant in terms of contributing to cell adhesion and migration during immune responses, differences were observed in the different immune cells. Elevated CD11b expression is often associated with the activation and mobilization of immune cells, particularly in response to infection or inflammation [30]. For instance, in lymphocytes, there is a clear and significant decrease in the percentage of cells expressing the molecule in individuals who consumed product B, and a non-significant increase in those who consumed product C. All three products decrease the density of molecules per lymphocyte, but the reduction is significant only in products A and C. In monocytes, there is no difference after supplementation in the percentage of CD11b+ monocytes. On the other hand, there is a clear and significant reduction in the density of CD11b per monocyte in those who consumed product C. Finally, in granulocytes, although the percentage of expression is not altered with any of the three supplementations, individuals who consumed products A and C saw a significant reduction in CD11b density per granulocyte.

In terms of joint expression of HLA-DR and CD11b, the supplementation with product C significantly increases the number of double-positive lymphocytes for these two molecules, but the comparison does not reach statistical significance. There are no differences in monocytes and granulocytes. 

### 3.3. Immunochemical Variables

Nutrition plays a crucial role in supporting the immune system, and this includes the production and function of immunoglobulins. Immunoglobulins, also known as antibodies, are proteins produced by the immune system to help defend the body against harmful pathogens like bacteria, viruses, and other microorganisms. In relation to food intake, the majority of the immune system cells and molecules are located in the gut-associated lymphoid tissue. A healthy gut microbiome, supported by a diet rich in fiber and probiotics, can positively influence the immune response, including the production of immunoglobulins [31]. The relationship between yogurt ingestion and immunoglobulins is often associated with the impact of probiotics on gut health. It is important to note that individual responses to yogurt and probiotics can vary. Factors such as the specific strains of bacteria in the yogurt, the overall diet, and an individual’s health status and habits can influence the outcomes. Additionally, more research is needed to fully understand the mechanisms by which yogurt and probiotics impact immunoglobulin production [32]. In this study, the effect of the relative amount of living bacteria is assessed. 

As can be observed in Table 2, the amount of serum IgG remains unchanged during supplementation with the three types of yogurts. Similarly, serum IgA concentration also remains unchanged during supplementation in all three groups of individuals, although there is a significant decrease in group A (pasteurized yogurt after fermentation) in the third extraction. The decrease is recovered in the fourth extraction and is close to the baseline. These results indicate that whether the bacteria have been killed or not does not necessarily impact the secretion of these IgG and IgA. Different results have been obtained; for instance, the evaluation of how consuming yogurt fermented with *Lactobacillus delbrueckii* ssp. *bulgaricus* OLL1073R-1 (1073R-1-yogurt) could affect influenza virus-bound salivary IgA levels in the elderly residents of nursing homes, as in this double-blind, parallel-group, randomized controlled trial, our results indicated an increase in these IgA levels. The health status of the individuals, especially if the immune system is compromised, has an impact on how nutrition immunomodulates the responses [33].

On the other hand, the changes in the serum concentration of IgM are interesting. IgM is the immunoglobulin involved in primary immune responses. A clear decrease in serum levels can be seen in both group A and group C, with the latter being more prominent, and an increase in group B when we compare extractions three and four with the initial one (Table 2). The decrease in IgM levels in consumers of A and C is significant compared to the third extraction. When observing the stratified data, in group A, the decrease was only observed in women, while it was also seen that the tendency to increase IgM of total group B is based on women between 20 and 45 years of age (data available upon request). In the context of nutrition and fermented foods, there is limited direct evidence on the impact of IgM levels in response to the ingestion of living or dead bacteria [34]; thus, these results represent preliminary evidence that the status of bacteria (based on the heat treatment applied) would impact the health benefits of yogurt.

The amount of total IgE in serum is also significantly affected by supplementation. Product A during the “run-in” (from day −14 to day 0) induces a significant increase. In the subsequent randomization, the individuals in groups B and C maintained similar levels at the end of the run-in, but on the other hand, the individuals in group A continue to see their levels increase significantly throughout the study period (Table 1). Stratification by age revealed that the differences between the three groups are at the maximum in individuals over 60 years of age (data available upon request). IgE is an antibody class associated with allergic responses and plays a central role in the immune system’s reaction to certain allergens, including those derived from foods. While IgE is not typically associated with responses to beneficial bacteria found in fermented foods, the relationship between IgE and bacterial ingestion can be influenced by various factors, including the specific context and individual immune responses. In this line, for instance, Hong and Chen [35] showed that consuming heat-inactivated *L. kefiranofaciens* M1 isolated from kefir grains could inhibit IgE production after an in vivo ovalbumin exposure in a mouse allergy model.

In addition, the amount of complement factor C3 decreases significantly in individuals who consumed product B. In any case, this significance must be downplayed because in groups A and C a decrease in the MFI is also observed, which is not significant due to the dispersion of the data. Specific mechanisms and outcomes can vary based on the types of bacteria present in fermented foods, the individual’s immune status, and the overall diet. Finally, the quantification of C-reactive protein is not altered with any of the three dietary supplements, indicating no effects of bacteria (alive or not) in this parameter. In fact, CRP is a non-specific marker of inflammation, meaning it can be elevated in response to various inflammatory stimuli, including infections, tissue injury, and autoimmune conditions. According to the literature, in the context of bacterial infections, both live and dead bacteria can trigger an inflammatory response, leading to an increase in CRP levels [36].

Regular consumption of pasteurized yogurt that retains a detectable number of viable LABs (group B) behaves in an intermediate way to the other two exposed: The consumption of this fresh yogurt (group A) mainly enhances the adaptive immune response by increasing systemic levels of IgM (essential antibodies in the primary immune response). On the other hand, consumption of pasteurized yogurt without any viable bacteria (group C) by healthy adult individuals leads to a decrease in the amount of circulating IgM. Thus, IgM serum concentration is highly dependent on the quantity of viable bacteria consumed.

### 3.4. Phagocytosis and Burst

Immune cells, such as monocytes and granulocytes, which are involved in phagocytosis, depend on nutrition to survive and function properly. The process by which these cells take in and break down foreign particles, such as bacteria and other diseases, is known as phagocytosis. Regarding the phagocytic capacity, as reported in Table 3, in general, no changes were observed in the percentage of granulocyte phagocytosis, although the three supplementations significantly reduced the phagocytic capacity of these cells (the MFI is proportional to the number of phagocytosed bacteria). The percentage of phagocytic cells within monocytes is reduced equally in the three supplementations with respect to the initial conditions. In this function, the most interesting aspect is the phagocytosis index: phagocytosis after activation/basal phagocytosis. The three supplementations enhance this index, both for the total number of cells and for the approximation of the number of bacteria per cell, but this increase only reaches statistical significance for the individuals who consumed products A and B, and not those who consumed product C. An increase may suggest a positive impact on the innate immune system. It could mean that the intervention, in this case, has enhanced the ability of the immune cells to engulf and eliminate foreign particles. However, considering the results obtained, the magnitude of change seems not to have a meaningful impact on immune function and overall health [37]. Results found in the literature regarding heat-killed bacteria are scarce. For instance, recently, it was reported that heat-killed probiotics, including *Lactiplantibacillus plantarum* KC3 (LP3) and CKDB008 (LP8), and *Limosilactobacillus fermentum* SRK414 (LF4) could promote the clearance of foreign pathogens by enhancing the phagocytosis of macrophages [38]. Further investigations are needed to describe how specific microorganisms, heat-killed or not, have an impact on phagocytosis.

In relation to the oxidative shock (Table 4), in general, no significant changes are seen in the percentage or intensity of oxidative shock, both in granulocytes and monocytes, although there is a certain tendency towards a decrease in said MFI. Once again, the most interesting parameter within this functionality is the oxidative shock index in granulocytes and monocytes. The three groups of individuals have increased it, being significant for those who consumed products B and C at the end of the study.

### 3.5. NK Cytotoxic Activity

Natural Killer (NK) cells are a type of cytotoxic lymphocyte that play a critical role in the innate immune system, targeting and eliminating infected or abnormal cells, including cancer cells [39]. As can be observed in Table 5, during the “run-in” period, there is a clear decrease in NK activity, although it does not reach statistical significance. Individuals who consumed product C have better NK activity overall (both at the 10:1 and 5:1 ratio), although in general, there is a significant decrease in said activity with the three supplementations when compared to the initial moment. Nonetheless, the stratification, although it does not reach significance, showed that supplementation A decreases NK activity (10:1 and 5:1) mainly in men under 45 years of age (data available upon request).

In the same line as this study, Makino et al. [40] demonstrated enhanced NK cell activation by exopolysaccharides derived from yogurt fermented with *Lactobacillus delbrueckii* ssp. *bulgaricus* OLL1073R-1 in mice. This strain was also the only strain that induced the production of IFN-γ in vitro. The authors indicated that NK cell activation by OLL1073R-1 yogurt is EPS-dependent, occurs via IL-12- and IL-18-mediated IFN-γ production, and requires myeloid differentiation factor 88. However, these results are not carried out in humans and the differences in metabolism hinder extrapolation and comparison of results.

### 3.6. IFN-y Inducible Gene Expression

There was a very large dispersion of the data, which was not corrected when normalizing by the beta-actin content or when applying the logarithm of said normalization. Therefore, the authors consider it risky to draw conclusions regarding possible changes in the expression of the OAS-1, TLR-7, TLR-8, and TLR-9 genes, although they might be available upon request. To the authors’ knowledge, there is not a direct and well-established link between interferon-gamma (IFN-γ)-inducible gene expression and the ingestion of yogurt. IFN-γ is a cytokine involved in the immune response, and its inducible genes play a role in various cellular processes. The probiotics contained in the study products could influence gut microbiota, leading to changes in the immune system, but the specific relationship with IFN-γ inducible gene expression is not clearly defined, as scarce literature has investigated this [41,42]. Moreover, we have found an HLA-DR expression increase after yogurt consumption, and HLA-DR protein expression is also inducible by IFN-γ and, thus, can be considered as an IFN-γ-mediated induction.

### 3.7. Intracellular Cytokine Profile

CD69 expression serves as a crucial early activation marker on immune cells, particularly T cells, providing valuable insights into the rapid response and functional activation of the immune system in various experimental and clinical contexts [43]. Regarding the CD69 expression (Table 6) after stimulation of lymphocytes with PHA or CD3+CD28, all three supplementations appear to decrease basal CD69 expression (in unstimulated lymphocytes). On the other hand, after stimulating with PHA, no differences are seen with respect to the initial moment of the study, for any of the supplementations. On the contrary, when the cells are stimulated with the anti-CD3 + anti-CD28 cocktail, there is a marked decrease in the expression of CD69 in the individuals who consumed product C, although it is not statistically significant. Furthermore, in the stratification of the sample, it is seen that this decrease in group C fundamentally affects women over 30 years of age.

On the other hand, the intracellular synthesis of interferon-gamma is crucial for activating antiviral mechanisms, coordinating immune responses, regulating inflammation, and contributing to immunosurveillance against tumors, highlighting its pivotal role in immune defense and homeostasis [44]. In this regard, the basal production of interferon-gamma (in unstimulated cells) increases in all three groups. However, the expression also increases after stimulation with PHA, being significant at the end of the study both for individuals who consumed product B and for those who consumed product C. Continued consumption of yogurt (any of the three) also increases the expression of CD69 induced by anti-CD3 + anti-CD28, but it is only significant in the case of fresh yogurt (B). When both parameters (CD69+ and IFN-gamma+) are combined, significant differences are observed in PHA-stimulated lymphocytes, but these differences cannot be taken into account since there is a large dispersion of data.

### 3.8. Cytokine Production

The production of cytokines, which are signaling proteins involved in immune responses, is intricately linked to nutrition. Nutritional factors play a crucial role in modulating the production and balance of cytokines, influencing immune function. The potential effects that yogurt may have on cytokine production stem from the bacteria, influencing the gut microbiota, which may impact the regulation of cytokine production, as well as the content of micronutrients such as zinc or vitamins. Some studies suggest that the consumption of probiotics, like those found in yogurt, may enhance anti-inflammatory cytokine production while downregulating pro-inflammatory cytokines [45,46]. Measuring cytokines following anti-CD3 + anti-CD28 stimulation is a powerful tool for understanding T cell activation and function. In the Supplementary Material—Table S3, the results of cytokine production when stimulated with CD3+CD28 are presented. 

No relevant differences between groups were observed in IL-6 secretion either in unstimulated or stimulated lymphocytes. However, when calculating the index (stimulated/unstimulated) it is observed that the three types of yogurts induce an increase in the secretion of IL-6 compared to the baseline situation before the intervention. There are no differences in IL-8 synthesis in inactivated lymphocytes between the three supplementation groups. On the other hand, when activated with the antibody cocktail, there is a significant increase in the synthesis of IL-8 compared to baseline, but there is no difference between supplementations, so it seems that all of them exert the same action. In the case of IL-10, there are no differences in its synthesis between groups (neither in unstimulated nor antibody-stimulated lymphocytes). In this case, the synthesis index (activated/inactivated) is significantly increased in all cases, and there are no differences between supplementations. 

There were no changes in the pattern of synthesis, neither in unstimulated cells, nor in stimulated cells, nor in the synthesis index, though there were significant differences between the three supplementation groups for IL-2 and IFN-gamma. In the case of IL-12, the synthesis was in many cases null, and when not, the data are very dispersed so no conclusions can be drawn. For TNF-α secretion, there is a clear trend towards increased synthesis after anti-CD3 + anti-CD28 stimulation. However, the changes observed between groups do not reach clear significance due to the dispersion of data. The stimulated/unstimulated ratio is not helpful either.

Results found in the literature regarding heat-killed bacteria are scarce. Recently, it was reported that heat-killed probiotics, including *Lactiplantibacillus plantarum* KC3 (LP3) and CKDB008 (LP8), and *Limosilactobacillus fermentum* SRK414 (LF4), could induce the production of NO and pro-inflammatory cytokines, including TNF-α, IL-6, and IL-1β [38]. However, this in vitro result lacks biological relevance in the context of human health.

In the case of the secretion of IL-1β, at baseline (unstimulated cells) synthesis is similar in the three groups and lower than the baseline situation (pre-supplementation). In the stimulated cells, there is a clear induction of IL-1β synthesis, but it is indistinguishable from supplementation. The index corroborates this as it increases significantly. In addition to that, in both unstimulated and antibody-stimulated cells, it is observed that individuals supplemented with product B reduce their production of IL-5, while individuals supplemented with products A or C increase it. However, this clear trend is not statistically significant. Finally, there are no clear changes in the pattern of IL-4 synthesis. Neither in unstimulated cells, nor in stimulated cells, nor in the rate of synthesis were there any significant discordant differences between the three supplementation groups.

### 3.9. Limitations of the Study

In Table 7, a summary of all the relevant changes based on the different group outcomes is presented. However, the authors are aware that the experimental design of the study has some limitations, which prevents more solid conclusions from being drawn and which must be considered in future studies.

- The absence of a control group (white) that had not eaten yogurt of any kind for 8 weeks.
- The small number of individuals in the group that consumed PMI pasteurized yogurt (group C), which resulted in notable changes in some variables not reaching statistical significance.
- The absence of analysis months after having completed the dietary intervention, which would have allowed us to see if the immunomodulation observed is transitory or remains over time.

## 4. Conclusions

This study included an extensive panel of in vitro indexes of immune function and has shown mild evidence of an enhanced immunostimulatory effect of the routine intake of fresh versus heat-treated yogurt by oral route. In particular, more mechanisms of the adaptative immune system are enhanced with the presence of relevant amounts of viable bacteria, while the innate immune system could be also modulated by the presence of heat-killed bacteria.

With the data obtained in the studied population, the starting hypothesis of the work is accepted: the consumption of fresh yogurt (and not pasteurized yogurt) favors the synthesis of IFN-gamma and some proteins inducible by IFN-gamma, as the density of HLA-DR expression on group B. However, no increase in genes inducible by IFN-gamma (OAS or TLRs) has been found. Regular and prolonged consumption of yogurt (with live or pasteurized bacteria) is beneficial for the immune system of healthy adult individuals. In particular, for different mechanisms of innate immunity, it promotes phagocytic activity, increasing the phagocytosis capacity of granulocytes, as well as the intracellular degradation capacity of the bacteria, as well as through the use of adapter molecules, increasing the cells that express CD16. Also, it promotes the synthesis of IL-8, relevant in the process of leukocyte extravasation, and of proinflammatory cytokines, such as IL-6 or IL-1 β, both normally produced in the activation of monocytes-macrophages. On top of that, the consumption of this fresh yogurt mainly enhances different mechanisms of the specific or adaptive immune response, including an increase in systemic levels of IgM, essential antibodies in the primary immune response. Additionally, regarding T lymphocytes, it favors the development of Th1 profiles (increasing the synthesis of IFN-gamma), inhibits Th2 (decreasing the production of IL-5), and facilitates an enhancement of the development of T lymphocytes in peripheral blood (mainly “naïve”-type helpers with a decrease in cytotoxic T lymphocytes) and slightly decreases NK lymphocytes. Finally, together with the already described outcomes, regular and prolonged consumption of pasteurized yogurt without viable bacteria by healthy adult individuals also potentiates mostly new innate mechanisms and maintains an activated circulating cellular phenotype or memory, including an increase in the number of circulating NK lymphocytes (CD16, CD56 and CD11b) and their cytotoxic function, a decrease in T cell activation in response to PHA, an increase in the synthesis of IL-5, which translates into a Th2 response, as well as a decrease in the amount of circulating IgM and of T lymphocytes (mainly naïve cooperators), together with an increase in B lymphocytes. Also, we observed an increase in the expression of CD45R0 and HLA-DR, markers of memory/lymphocyte activation. However, a more potent increase in adaptative immune mechanisms is only obtained after the consumption of fresh (live bacteria) yogurt, as demonstrated in these studies.

Research in the field of immunonutrition is ongoing, and newer studies may provide more insights into the specific impacts of yogurt and probiotics on immune cell subpopulations. It is essential to note that individual responses can vary, and other factors like the overall diet, lifestyle, and health status also play crucial roles in immune function.

## Figures and Tables

**Table 1:** Leukocyte and lymphocyte subpopulations.

		Day			
Parameter	Group	−14	0	21	42
% CD3+ lymphocytes	A	74.56 ± 7.05	75.48 ± 6.99	76.85 ± 6.58	76.24 ± 6.92
	B			75.17 ± 7.07	75.89 ± 6.88
	C			72.52 ± 6.69 *	73.29 ± 7.3 *
CD3+ lymphocytes/μL	A	1916.22 ± 583.63	1934.56 ± 584.23	1998.67 ± 669.54	1982.56 ± 675.94
	B			1934.4 ± 536.02	1973.83 ± 523.42
	C			1784.04 ± 518.14 *	1803.02 ± 502.75 *
% CD3+ CD4+ T helper lymphocytes	A	46.37 ± 7.51	47.7 ± 7.28	48.5 ± 7.29	48.09 ± 6.9
	B			48.38 ± 7.73	49.15 ± 7.5 *
	C			45.08 ± 6.62	45.97 ± 7.88 *
% Cytotoxic T lymphocytes CD3+ CD8+	A	22.96 ± 8.68	22.82 ± 7.72	23.81 ± 8.34	23.88 ± 7.56
	B			21.53 ± 7.52	21.68 ± 7.29
	C			22.16 ± 8.02	22.45 ± 7.77
Ratio of CD4+/CD8+ helper/cytotoxic T lymphocytes	A	2.33 ± 1.02	2.39 ± 1.04	2.35 ± 1.02	2.28 ± 0.95
	B			2.58 ± 1.17 *	2.58 ± 1.1 *
	C			2.37 ± 1.08	2.38 ± 1.12
% B lymphocytes CD19+	A	9.92 ± 3.14	10.07 ± 3.15	10.2 ± 3.11	10.41 ± 2.92
	B			10.51 ± 3.39	10.56 ± 3.53
	C			11.07 ± 3.38 *	10.98 ± 3.48 *
Ratio of T/B CD3+/CD19+ lymphocytes	A	8.44 ± 3.14	8.38 ± 3.33	8.65 ± 4.61	8.37 ± 4.77
	B			8.01 ± 3.03	7.95 ± 2.59
	C			7.22 ± 2.41	7.47 ± 3.03
% NK CD3− CD56+ CD16+ lymphocytes	A	14.97 ± 10.88	13.87 ± 6.71	12.27 ± 5.68 *	12.65 ± 5.81
	B			13.75 ± 7.36 *	12.86 ± 7.18
	C			15.73 ± 5.6 *	15.14 ± 6.99 *
% Naïve T lymphocytes CD3+ CD45RA+ CD45R0−	A	30.56 ± 11.13	30.53 ± 11.21	30.56 ± 11.17	31 ± 10.43
	B			32.61 ± 11.55	33.47 ± 11.67
	C			25.51 ± 10.5 *	26.36 ± 10.66 *
% Memory T lymphocytes CD3+ CD45RA− CD45R0+	A	35.86 ± 12.86	34.4 ± 12.19	33.57 ± 12.64 *	34.32 ± 12.43
	B			32.64 ± 11.18	34.34 ± 11.08
	C			36.92 ± 12.04	39.35 ± 14.04
Ratio of naïve T lymphocytes to memory	A	1.12 ± 0.71	1.07 ± 0.67	1.11 ± 0.67	1.17 ± 0.88
	B			1.23 ± 0.81	1.18 ± 0.73
	C			0.83 ± 0.55 *	0.92 ± 0.89 *
% Naïve helper T lymphocytes CD3+ CD4+ CD45RA+ CD45R0−	A	13.70 ± 7.63	13.7 ± 7.61	13.44 ± 7.13	14.04 ± 7.63
	B			14.97 ± 8.1	16.17 ± 8.44
	C			11.22 ± 6.38	11.96 ± 6.44
%Memory helper T lymphocytes CD3+ CD4+ CD45RA-R0+	A	26.65 ± 9.35	25.51 ± 9.67	24.93 ± 9.12	25.53 ± 9.52
	B			25.27 ± 8.82	26.75 ± 8.62
	C			25.96 ± 9.67	28.12 ± 11.8 *
Ratio of naïve helper T cells to memory	A	0.64 ± 0.40	0.64 ± 0.51	0.62 ± 0.4	0.66 ± 0.48
	B			0.73 ± 0.57	0.72 ± 0.52
	C			0.51 ± 0.36	0.57 ± 0.5
% Naïve cytotoxic T lymphocytes CD3+ CD8+ CD45RA+ CD45R0−	A	17.69 ± 6.90	17.82 ± 7.12	17.75 ± 7.1	17.79 ± 6.25
	B			18.31 ± 7.15	17.96 ± 7.3
	C			15.21 ± 6.11 *	15.18 ± 6.74 *
MFI naïve cytotoxic T lymphocytes CD3+ CD8+ CD45RA+ CD45R0−	A	15.73 ± 4.52	14.02 ± 4.42	12.76 ± 4.38	12.63 ± 3.43
	B			12.5 ± 3.36	12.74 ± 2.7
	C			12.28 ± 3.74	12.04 ± 2.84
% Cytotoxic T lymphocytes memory CD3+ CD8+ CD45RA− CD45R0+	A	10.14 ± 5.89	9.59 ± 5.14	9.33 ± 5.13	9.72 ± 5.25
	B			8.12 ± 4.04	8.33 ± 3.83
	C			11.89 ± 7.87 *	12.11 ± 7.34 *
Ratio of naïve cytotoxic T lymphocytes to memory	A	2.79 ± 1.48	2.57 ± 2.04	2.75 ± 2.14	3.11 ± 3.66
	B			2.95 ± 1.97	2.67 ± 1.69
	C			1.88 ± 1.45 *	1.96 ± 1.87 *
% CD11b Expression in Lymphocytes	A	33.03 ± 9.38	31.75 ± 8.89	30.13 ± 9.54	31 ± 8.72
	B			30.7 ± 8.23	30.25 ± 8.43
	C			34.92 ± 8.35 *	34.43 ± 11.42 *
% HLA-DR and CD11b Expression in Lymphocytes	A	6.51 ± 3.11	6.46 ± 2.71	6.45 ± 2.23	6.48 ± 1.89
	B			6.5 ± 2.36	6.6 ± 2.84
	C			8.03 ± 3.35 *	8.07 ± 3.2 *
% HLA-DR Expression in Monocytes	A	53.57 ± 16.90	53.37 ± 17.19	50.83 ± 16.85 *	49.62 ± 16.58 *
	B			54.24 ± 14.33	53.14 ± 16.06
	C			55.02 ± 18.7	54.96 ± 19.41
% CD11b Expression in Monocytes	A	99.85 ± 0.33	99.9 ± 0.12	99.87 ± 0.16	99.86 ± 0.14
	B			99.86 ± 0.18	99.85 ± 0.2
	C			99.85 ± 0.17	99.91 ± 0.14
MFI CD11b expression in monocytes	A	96.64 ± 16.90	91.91 ± 21.62	89.69 ± 18.7	91.73 ± 23.01
	B			89.73 ± 17.24	90.27 ± 22.01
	C			84.26 ± 21.33 *	84.84 ± 17.37 *
% CD11b Expression in Granulocytes	A	100.00 ± 0.01	100.00 ± 0.01	100.00 ± 0.01	100.00 ± 0.01
	B			100.00 ± 0.01	100.00 ± 0.01
	C			100.00 ± 0.01	100.00 ± 0.01
MFI CD11b expression in granulocytes	A	80.96 ± 11.46	83.21 ± 70.11	74.74 ± 14.6 *	75.63 ± 14.89 *
	B			79.96 ± 16.76	77.34 ± 19 *
	C			73.18 ± 13.65 *	73.31 ± 16.62 *

MFI: mean fluorescence intensity. * Denotes statistically significant differences between the group compared to the day −14.

**Table 2:** Serum immune proteins from before (day −14) to the end of the intervention (day 42).

		Day			
Parameter	Group	−14	0	21	42
IgG (mg/dL):	A	1031.46 ± 162.08	1059.1 ± 205.53	1031.25 ± 148.55	1038.96 ± 158.06
	B			1054.02 ± 246.8	1068.94 ± 223.82
	C			1056.24 ± 213.66	1058 ± 205.65
IgA (mg/dL)	A	205.37 ± 69.52	217.89 ± 77.61	208.54 ± 75.74	213.47 ± 75.07
	B			214.81 ± 78.03	216.4 ± 68.41
	C			220.8 ± 75.43	218.99 ± 80.27
IgM (mg/dL):	A	121.54 ± 55.21	136.07 ± 68.75	128.67 ± 58.39	128.16 ± 60.72
	B			141.81 ± 66.49	140.67 ± 66.3
	C			109.07 ± 52.15 *	106.09 ± 53.68 *
IgE total (KU/L):	A	24.52 ± 8.56	30.43 ± 7.89	37.18 ± 9.95	42.76 ± 9.61
	B			32.35 ± 9.96	33.79 ± 8.84
	C			30.37 ± 8.62	29.04 ± 7.59
C3c (mg/dL):	A	121.89 ± 19.86	124.6 ± 25.29	118.15 ± 21.31	119.5 ± 22.9
	B			121.04 ± 28.18	119.64 ± 27.82
	C			121.96 ± 18.68	116.12 ± 16.57
PCR (mg/L):	A	0.81 ± 1.2	1.98 ± 5.8	1.07 ± 1.03	1.4 ± 1.92
	B			1.49 ± 2.04	1.42 ± 1.77
	C			1.83 ± 3.31	2.17 ± 4.13

* Denotes statistically significant differences between the group compared to the day −14.

**Table 3:** Phagocytosis—Percentage and MFI bactericidal activity of granulocytes and monocytes from before (day −14) to the end of the intervention (day 42).

		Day			
Parameter	Group	−14	0	21	42
% and (MFI) Granulocyte phagocytosis/in the presence of *E. coli*	A	91.71 ± 9.59 (476.33 ± 158.0)	93.43 ± 6.95 (401.34 ± 147.75)	91.21 ± 5.73 (328.41 ± 96.48 *)	89.44 ± 9.41 (322.51 ± 92.73 *)
	B			91.02 ± 6.01 (345.11 ± 110.36 *)	89.86 ± 7.41 (344.97 ± 117.8 *)
	C			91.46 ± 7.22 (334.68 ± 105.39 *)	91.64 ± 5.58 (350.93 ± 108.21 *)
% and (MFI) Phagocytosis of monocytes in the presence of *E. coli*:	A	80.16 ± 12.49 (228.32 ± 60.75)	80.06 ± 11.93 (206.2 ± 61.02)	72.69 ± 11.08 * (189.49 ± 40.2 *)	74.65 ± 10.87 * (198.32 ± 46.16 *)
	B			70.34 ± 10.19 * (198.56 ± 48.44 *)	73.85 ± 9.89 * (209.26 ± 48.13 *)
	C			74.08 ± 10.01 * (201.54 ± 45.23 *)	74.64 ± 10.76 * (206.75 ± 41.35 *)
Indices % and (MFI) Phagocytosis in granulocytes	A	57.38 ± 42.12 (16.76 ± 11.12)	55.42 ± 35.45 (18.21 ± 9.44)	57.19 ± 42.07 (15.74 ± 6.76)	60.63 ± 32.69 (17.81 ± 6.04)
	B			56.30 ± 32.79 (14.11 ± 6.39)	64.44 ± 28.58 (18.79 ± 10.01)
	C			56.49 ± 27.45 (15.55 ± 6.77)	76.24 ± 42.75 * (17.96 ± 8.45)
% and (MFI) phagocytosis rates in monocytes	A	33.43 ± 22.36 (7.91 ± 3.48)	29.70 ± 19.18 (8.67 ± 3.55)	32.41 ± 23.29 (8.98 ± 3.53)	42.55 ± 50.29 * (10.11 ± 2.93 *)
	B			38.69 ± 37.98 * (7.66 ± 2.67)	42.85 ± 38.90 * (10.24 ± 3.3 *)
	C			35.22 ± 27.98 (8.78 ± 3.55)	35.43 ± 21.4 (9.51 ± 3.13 *)

MFI: mean fluorescence intensity. * Denotes statistically significant differences between the group compared to the day −14.

**Table 4:** Burst—Percentage and MFI bactericidal activity of granulocytes and monocytes from before (day −14) to the end of the intervention (day 42).

		Day			
	Group	−14	0	21	42
% and (MFI) Granulocyte Burst in the Presence of *E. coli*:	A	94.57 ± 9.10 (250.09 ± 88.75)	96.75 ± 4.43 (228.4 ± 85.38)	95.19 ± 6.32 (215.5 ± 67.51)	96.58 ± 4.26 (227.51 ± 70.27)
	B			95.28 ± 4.41 (207.9 ± 65.38)	96.75 ± 3.88 (228.45 ± 65.23)
	C			94.97 ± 6.42 (209.78 ± 85.4)	96.47 ± 3.85 (213.39 ± 51.67)
% Burst and (MFI) monocytes in the presence of *E. coli*	A	69.38 ± 19.22 (79.10 ± 33.88)	69.66 ± 18.59 (67.62 ± 26.72)	66.99 ± 18.54 (74.09 ± 36.69)	66.72 ± 16.97 (69.35 ± 28.13)
	B			64.73 ± 16.76 (72.16 ± 20.41)	67.04 ± 13.32 (68.1 ± 17.58)
	C			66.94 ± 15.91 (72.33 ± 32.92)	63.64 ± 11.86 (83.3 ± 102.65)
% and (MFI) Burst indices in granulocytes	A	17.11 ± 7.91 (5.23 ± 2.74)	24.17 ± 13.18 (5.29 ± 2.33)	23.12 ± 13.82 * (5.78 ± 2.59)	19.25 ± 11.68 (6.63 ± 2.44)
	B			23.55 ± 14.84 * (5.43 ± 2.07)	21.83 ± 12.35 * (6.31 ± 2.18)
	C			24.62 ± 15.34 * (5.54 ± 3.09)	24.47 ± 15.79 * (5.57 ± 1.59)
% and (MFI) Burst indices in monocytes	A	12.70 ± 6.34 (1.63 ± 0.38)	17.88 ± 22.77 (1.58 ± 0.74)	16.47 ± 9.59 * (1.9 ± 1.34)	11.41 ± 5.49 (1.81 ± 0.51)
	B			13.35 ± 9.49 (1.8 ± 0.61)	12.72 ± 8.77 (1.76 ± 0.27)
	C			18.77 ± 6.42 * (1.78 ± 0.47)	11.81 ± 6.21 (2.04 ± 2.08)

MFI: mean fluorescence intensity. * Denotes statistically significant differences between the group compared to the day −14.

**Table 5:** Averages of NK cytotoxic activity from before (day −14) to the end of the intervention (day 42).

		Day			
Parameter	Group	−14	0	21	42
% lysis of K562 cells by NK cells in a 10:1 (and 5:1) ratio	A	22.41 ± 9.23	17.50 ± 8.73 (13.24 ± 6.6)	17.71 ± 7.78 * (14.03 ± 6.49)	17.30 ± 7.44 * (13.97 ± 5.85)
	B			21.13 ± 9.14 (16.08 ± 7.04)	19.11 ± 10.32 * (16.03 ± 8.79)
	C			19.76 ± 6.73 * (16.68 ± 6.7)	21.43 ± 8.85 (18.06 ± 8.62)
% spontaneous lysis of K562 cells	A	4.04 ± 5.97	3.46 ± 2.14	5.09 ± 3.31	5.34 ± 2.85
	B			5.24 ± 3.37	5.69 ± 3.01 *
	C			5.29 ± 3.47	5.39 ± 3.07
% total K562 cell lysis	A	93.81 ± 7.85	92.22 ± 13.17	97.18 ± 2.65 *	96.73 ± 3.05 *
	B			97.34 ± 2.74 *	96.69 ± 2.99 *
	C			97.56 ± 2.45 *	97.05 ± 2.91 *
NK Activity 10:1 (and 5:1) corrected (%)	A	20.84 ± 10.40 (13.64 ± 9.39)	16.31 ± 9.68 (11.23 ± 7.4)	14.79 ± 7.3 * (9.64 ± 7.05 *)	14.16 ± 7.1 * (9.45 ± 6.13 *)
	B			17.07 ± 9.89 * (11.64 ± 7.45)	15.89 ± 10.8 * (11.35 ± 9.61)
	C			15.58 ± 8.07 * (12.22 ± 8.03)	17.5 ± 8.74 * (13.8 ± 8.45)

* Denotes statistically significant differences between the group compared to the day −14.

**Table 6:** Intracellular cytokine profile.

		Day			
	Group	−14	0	21	42
% CD69 Expression in PHA-Stimulated Cells	A	31.37 ± 11.08	31.00 ± 11.14	28.50 ± 12.09	29.83 ± 13.37
	B			31.47 ± 9.04	30.59 ± 14.07
	C			30.21 ± 11.57	27.44 ± 10.21
% IFN-gamma expression of PHA-stimulated cells	A	17.13 ± 15.09	18.33 ± 14.5	20.62 ± 17.8	20.26 ± 17.57
	B			17.59 ± 12.16	20.50 ±16.94
	C			15.45 ± 12.12	24.32 ± 17.93 *
% CD69/IFN-gamma expression of PHA-stimulated cells	A	3.27 ±3.25	2.21 ± 1.88	1.52 ± 1.45 *	1.82 ± 1.88 *
	B			1.45 ± 0.96 *	1.97 ± 1.94
	C			1.81 ± 1.52 *	1.74 ± 1.39 *
% MFI	A	11.70 ± 5.41	11.25 ± 5.36	9.01 ± 3.05 *	9.08 ± 2.99 *
	B			9.15 ± 3.11 *	8.85 ± 2.86 *
	C			8.85 ± 3.06 *	9.20 ± 2.75 *
% MFI IFN-gamma Expression of PHA-Stimulated Cells	A	4.44 ± 3.70	3.63 ±3.29	2.45 ± 1.19 *	2.57 ± 1.38
	B			2.55 ± 1.51	2.55 ± 1.19
	C			2.67 ± 1.15	2.47 ± 1.26 *
% CD69 expression of CD3+CD28-stimulated cells	A	42.82 ± 19.09	45.58 ± 18.12	44.92 ± 18.93	45.23 ± 19.6
	B			46.34 ± 19.14	45.98 ± 19.03
	C			38.97 ± 19.97 *	38.97 ± 20.09 *
% IFN-gamma expression of CD3+CD28-stimulated cells	A	14.47 ± 12.44	14.15 ± 12.26	16.54 ± 14.14	16.72 ± 13.81
	B			14.80 ± 10.79	18.27 ± 16.01 *
	C			12.28 ± 9.65	18.10 ± 12.97 *
% CD69/IFN-gamma expression of CD3+CD28-stimulated cells	A	4.78 ± 4.32	4.61 ± 4.26	3.96 ± 4.12	4.17 ± 3.25
	B			3.69 ± 3.15	4.45 ± 3.69
	C			3.23 ± 3.28 *	3.46 ± 2.85 *
% MFI CD69 Expression of CD3+CD28-Stimulated Cells	A	10.08 ± 4.98	9.00 ± 4.29	7.55 ± 2.34 *	8.00 ± 2.47
	B			7.75 ± 2.84	7.80 ± 2.4
	C			7.30 ± 2.39 *	7.62 ± 2.55 *
% MFI IFN-gamma expression of CD3+CD28-stimulated cells	A	4.55 ± 3.34	4.77 ± 4.3	3.30 ± 1.92	3.40 ± 1.76
	B			3.95 ± 3.34	3.62 ± 1.92
	C			3.35 ± 1.96	2.62 ± 1.11 *

MFI: mean fluorescence intensity. * Denotes statistically significant differences between the group compared to the day −14.

**Table 7:** Summary of the changes observed according to the supplementations.

A and C	B	A	C	A, B, C
↓ IgM ↑ IL-5 ↓ CD11b MFI	↑ IgM ↓ C3 ↑ IFN CD3/28 ↓ IL-5 ↑ CD3 ↑ CD4 ↓ CD8 ↑ CD4/CD8 ↑ CD3 RA ↑ DR MFI ↓ %CD11B lympho	↑ IgE ↑ CD4	↑ NK 10:1 ↓ CD69-PHA ↓ CD3 ↓ CD4 ↑ CD19 ↓ CD3/CD19 ↑ NK% ↓ CD3RA ↑ CD3R0 ↓ RA/R0 CD3 ↑ DR ↓ MFI ↑ %CD11B lympho	↑ Phagocytosis ↑ Burst ↑ IL-6 ↑ IL-8 ↑ IL-1beta ↑ CD16 ↓ MFI ↓ CD14 Lympho%; MFI monocyotes, MFI granulocytes ↓ CD14% ↓C D86

↑ and ↓ meaning increase and decrease respectively, compared to baseline.

## Data Availability

Data might be available upon request to the corresponding author.

## Supplementary Materials

The following supporting information can be downloaded at: https://www.mdpi.com/article/10.3390/nu16121969/s1, Table S1: Summary of routine hematology at baseline; Table S2: Summary of routine biochemistry at baseline; Table S3: Cytokine production following stimulation with CD3+CD28 from before (day −14) to the end of the intervention (day 42).
