# Supplementary material for: Prospective, Randomized, Double-Blind Parallel Group Nutritional Study to Evaluate the Effects of Routine Intake of Fresh vs. Pasteurized Yogurt on the Immune System in Healthy Adults
Source: Nutrients. 2024 Jun 20;16(12):1969. doi: 10.3390/nu16121969 (PMC11206341; doi:10.3390/nu16121969)
Supplement: Supplementary file 1 [file nutrients-16-01969-s001.zip › nutrients-3023992-supplementary.pdf]

Supplementary material

Table S1: Summary of routine hematology at baseline

Parameter	Pasteurized yogurt (n=48)	Fresh yoghurt (n=48)	Sterilized yogurt (n=25)
Erythrocytes (10^{18}/l)	4.8 ± 0.4	4.8 ± 0.4	4.8 ± 0.4
Hemoglobin (g/l)	14.6± 1.2	14.6± 1.3	14.5± 1.2
Hematocrit (%)	42.7± 3.3	42.8± 3.3	42.3± 3.6
Reticulocytes (‰.)	16.0 ± 4.4	15.8 ± 3.0	17.5 ± 5.7
Leukocytes (10^9/l)	6.8 ± 1.6	6.6 ± 1.4	6.2 ± 1.5
Neutrophils (10^9/l)	3.5 ± 0.9	3.4 ± 0.9	3.2 ± 1.0
Eosinophils (10^9/l)	0.2 ± 0.2	0.2 ± 0.1	0.2 ± 0.1
Basophils (10^9/l)	0.0 ± 0.0	0.0 ± 0.0	0.0 ± 0.0
Granulocytes (10^9/l)	3.8 ± 1.0	3.7 ± 1.0	3.4 ± 1.0
Lynphocytes (10^9/l)	2.6 ± 0.8	2.6 ± 0.6	2.5 ± 0.6
Monocytes (10^9/l)	0.4 ± 0.1	0.4 ± 0.1	0.3 ± 0.1
Platelets (10^6/l)	0.3 ± 0.1	0.2 ± 0.0	0.2 ± 0.0

Table S2: Summary of routine biochemistry at baseline

Parameter	Pasteurized yogurt (n=48)	Fresh yoghurt (n=48)	Sterilized yogurt (n=25)
Serum iron (µd/dl)	94.1 ±33.4	92.2 ±33.4	94.4 ±28.0
Transferrin (mg/dl)	265.8 ± 46.3	265.7 ± 29.2	255.4 ± 50.2
Glucose (mg/dl)	87.0 ± 10.2	89.5 ± 9.4	88.9 ± 9.7
Urea (mg/dl)	34.5 ± 7.8	36.1 ± 7.6	35.6 ± 7.8
Total cholesterol (mg/dl)	198.1 ± 35.2	198.9 ± 37.7	189.24 ± 37.2
HDL cholesterol (mg/dl)	67.7 ± 16.6	73.9 ± 17.0	65.5 ± 20.5
Triglycerides (mg/dl)	89.4 ± 58.5	82.0 ± 50.0	89.7 ± 27.0
Creatinine (mg/dl)	1.1 ± 0.2	1.1 ± 0.2	1.0 ± 0.2
Total bilirubin (mg/dl)	0.6 ± 0.2	0.6 ± 0.1	0.6 ± 0.1
Serum proteins (g/dl)	7.1 ± 0.4	7.0 ± 0.4	7.0 ± 0.5
Serum albumin (g/dl)	4.2 ± 0.2	4.3 ± 0.3	4.3 ± 0.3
Sodium (mEq/l)	140.6 ± 2.4	140.7 ± 2.5	140.6 ± 2.4
Potassium (mEq/l)	4.3 ± 0.3	4.3 ± 0.3	4.3 ± 0.3
Chloride (mEq/l)	103.9 ± 2.5	104.4 ± 2.7	104.9 ± 2.9
Glutamic oxaloacetic transaminase (IU/l)	22.4 ± 6.4	23.8 ± 7.7	26.4 ± 12.2
Glutamic pyruvic transaminase (IU/l)	20.5 ± 9.0	21.7 ± 8.3	28.7 ± 25.6

Table S3: Cytokine production following stimulation with CD3+CD28 from before (day -14) to the end of the intervention (day 42)

IL-6		Average	sd
0	Dia 0	4238,93	3297,95
21	Dia 21 - A	4426,68	2904,12
	Dia 21 - B	5262,11	3348,78
	Dia 21 - C	4912,23	3038,31
42	Dia 42 - A	3788,05	2546,59
	Dia 42 - B	3876,32	2576,6
	Dia 42- C	3819,78	2889,47
**IL-8**			
	Total	6640,58	4718,62
Day 21 (V3)	Grupo A	6974,37	3724,14
	Grupo B	8774,46	4686,87
	Grupo C	8931,59	5948,25
Day 42 (V4)	Grupo A	8766,44	3833,21
	Grupo B	8032,25	3969,98
	Grupo C	7870,55	4424,47
**IL-10**		x10E2	x10E2
	Total	124,19	125,68
Day 21 (V3)	Grupo A	158,01	138,2
	Grupo B	183,65	136,44
	Grupo C	174,03	176,29
Day 42 (V4)	Grupo A	149,9	147,60
	Grupo B	140,6	110,63
	Grupo C	143,1	134,02
**IL-2**			
	Total	1785,37	2533,06
Day 21 (V3)	Grupo A	1634,19	2142,2
	Grupo B	1580,13	1849,48
	Grupo C	1829,88	2648,92

Day 42 (V4)	Grupo A	1389,93	1876,9
	Grupo B	1787,7	2325
	Grupo C	1161,82	1213,21
IFN-gamma			
	Total	655,40	774,56
Day 21 (V3)	Grupo A	888,98	1085,4
	Grupo B	1149,65	1576,12
	Grupo C	1228,43	1719,57
Day 42 (V4)	Grupo A	750,42	745,9
	Grupo B	888,3	936
	Grupo C	661,43	925,03
IL-12p40			
	Total	144,89	701,56
Day 21 (V3)	Grupo A	0	0
	Grupo B	36,91	239,23
	Grupo C	0	0
Day 42 (V4)	Grupo A	3,1	16,53
	Grupo B	7,39	48,18
	Grupo C	6,92	33,91
TNF-alfa			
	Total	4028,12	9864,48
Day 21 (V3)	Grupo A	9514,04	20199,8
	Grupo B	4197,68	6899,82
	Grupo C	3305,29	4384,65
Day 42 (V4)	Grupo A	5734,21	9174,8
	Grupo B	4132,5	5753,46
	Grupo C	3792,56	5207,75
IL-1B			
	Total	1635,04	2146,5
Day 21 (V3)	Grupo A	1921,24	2023,7
	Grupo B	2048,79	2287,53
	Grupo C	2083,7	2135,66
Day 42 (V4)	Grupo A	2063	3015,68
	Grupo B	1536,24	1693,94
	Grupo C	2577	3014,8
IL-5			
	Total	78,53	141,17
Day 21 (V3)	Grupo A	73,99	108,16
	Grupo B	48,17	60,99

	Grupo C	121,4	273,37
Day 42 (V4)	Grupo A	75,08	108,97
	Grupo B	49,75	71,31
	Grupo C	80,06	161,94
IL-4			
	Total	443,19	772,12
Day 21 (V3)	Grupo A	361,37	540,9
	Grupo B	291,6	477,79
	Grupo C	1570,2	7219,07
Day 42 (V4)	Grupo A	265,38	426,59
	Grupo B	253,17	422,88
	Grupo C	198,18	287,26
